# Sugar-Sweetened Beverage (SSB) Intake Is Associated with Non-SSB Diet Quality in Swiss Adults

**DOI:** 10.3390/nu18050718

**Published:** 2026-02-24

**Authors:** Lukas Abraham, Flurina Suter, Giulia Pestoni, Sabine Rohrmann

**Affiliations:** 1 Division of Chronic Disease Epidemiology, Epidemiology, Biostatistics and Prevention Institute, University of Zurich, 8001 Zurich, Switzerland; 2 Cancer Registry Zurich, Zug, Schaffhausen and Schwyz, Institute of Pathology and Molecular Pathology, University Hospital Zurich, 8091 Zurich, Switzerland; 3 Nutrition Group, Swiss Distance University of Applied Sciences (FFHS)/University of Applied Sciences and Arts of Southern Switzerland (SUPSI), 8005 Zurich, Switzerland

**Keywords:** sugar-sweetened beverages, diet quality, dietary intake, public health

## Abstract

**Background/Objectives:** More than half of Swiss adults exceed the World Health Organization’s recommended limit for free sugar intake, with sugar-sweetened beverage (SSB) as a major contributor. SSB intake may be associated with other dietary risk factors, but little is known about diet quality excluding SSB intake. Therefore, the objective of this study was to investigate the association of SSB intake with the non-SSB diet quality in Swiss adults. **Methods:** Data from the cross-sectional, national nutrition survey *menuCH* (2014–2015, *n* = 2057, 18–75 years) were analyzed. Dietary intake was assessed via two 24 h dietary recalls. Participants were categorized as non-, low-, or high-SSB consumers based on the Swiss dietary guideline for free sugar intake. Diet quality excluding SSB was evaluated using the Alternative Healthy Eating Index (AHEI) scoring system (non-SSB AHEI). **Results:** Non-SSB consumers had higher non-SSB AHEI scores compared to low-SSB consumers, indicating healthier food choices beyond SSB intake, while high-SSB consumers had substantially poorer non-SSB diet quality. Despite these differences, non-SSB AHEI scores were only moderate across all SSB consumer types, suggesting that reducing SSB alone may not suffice to achieve optimal diet quality. **Conclusions:** In addition to population-based strategies to reduce SSB intake, future policies should aim to improve overall diet quality, including higher consumption of vegetables, fruits, whole grains and unsaturated fats. Prospective studies are needed to clarify which alternative food choices individuals make when reducing SSB intake.

## 1. Introduction

The prevention of non-communicable diseases (NCDs), such as obesity and cardiovascular diseases (CVD), is a priority of international health organizations and policymakers [1,2]. These diseases are the leading contributor to the Global Burden of Disease (GBD) [3] and healthcare costs of NCDs are rising significantly [4].

Sugar-sweetened beverage (SSB) consumption facilitates weight gain and contributes to the prevalence of obesity [5,6,7], which is a key risk factor for other NCDs [8]. Furthermore, evidence from large prospective cohort studies indicates that consumption of SSBs is directly associated with risk of CVD and type 2 diabetes [9]. Worldwide, an annually estimated 184,000 deaths were directly attributable to SSB consumption [10]. However, SSBs are not the only dietary component that has an influence on health. In a systematic analysis, the GBD 2017 Diet Collaborators found that diets high in sodium, low in whole grains, and low in fruits were the leading risk factors for deaths [11].

To quantify the healthfulness of dietary patterns, several validated diet quality indices exist [12,13,14,15,16]. The Alternative Healthy Eating Index (AHEI) was created based on food groups and nutrients that were shown to predict the risk of chronic diseases. It includes, among others, the above-mentioned dietary risk factors defined by the GBD 2017 Diet Collaborators. High diet quality assessed by the AHEI is associated with lower risk of CVD, cancer, and type 2 diabetes [17].

Research about the association between SSB intake and diet quality in adults is scarce and limited to populations from the US [18,19,20], where SSB intake is among the highest in the world [21]. Analyses from European countries, where different SSB consumption habits and diet quality were observed [22], are lacking.

The World Health Organization (WHO) recommends a limited free sugar intake of <10% of total energy intake [23]. In Switzerland, a multilinguistic country in Central Europe, over half of the adult population consumes more free sugars than recommended by the WHO [24]. Based on the Swiss National Nutrition Survey (*menuCH*), SSBs contribute 29% of total free sugar intake among Swiss adults and represent the main source of free sugars in the diet of young adults [24]. The Swiss Society for Nutrition recommends an optional intake of sweetened beverages, sweets, and salty snacks of 0–1 portions a day, with one portion of beverages (e.g., cola, energy drink, fruit juice) defined as 200 g, equivalent to approximately one small serving [25]. Data from *menuCH* show that the consumption of soft drinks and fruit juices alone exceeds this recommendation, with a mean daily intake of 299 g per person [26].

It remains unclear how SSB intake is related to diet quality, specifically whether differences in diet quality exist between individuals with high versus low SSB intake. We hypothesized that higher SSB intake would be associated with poorer diet quality, indicating a clustering effect of unhealthy dietary behaviors. Hence, the aim of this study was to investigate the association between SSB consumer type (non-, low-, and high-SSB consumers) and diet quality using *menuCH* data.

## 2. Materials and Methods

### 2.1. Study Design and Population

*menuCH* was a cross-sectional, population-based survey conducted from January 2014 to February 2015. It was the first and, to date, only nationally representative nutrition survey among adults in Switzerland. The Federal Statistical Office drew a stratified random sample of Swiss residents aged 18–75 years designed to be representative for both sexes, five age groups, the three main linguistic regions, and the 12 most populous Swiss cantons. Of 5496 eligible individuals who could be contacted by phone, 2086 (38%) agreed to participate. The final analytic sample comprised 2057 participants who fully completed the dietary assessment. A participant flowchart is provided in Figure S1. Detailed descriptions on the recruitment of *menuCH* participants have been published elsewhere [26,27,28].

### 2.2. Dietary Data

In the *menuCH* survey, trained dietitians obtained two non-consecutive 24 h dietary recalls (24HDR) from each participant. The first recall was conducted during an in-person interview and the second during a phone interview two to six weeks later. Dietary supplement intake was not assessed in the 24HDR. In our analyses, participants were only included if both 24HDRs were completed. Data collection was conducted over a period of one year, and interviews were evenly distributed across weekdays and seasons to account for seasonal and day-of-week variation. Dietary data was collected using the trilingual Swiss version (0.2014.02.27) of the internationally validated software GloboDiet^®^ (GD, formerly EPIC-Soft^®^, International Agency for Research on Cancer (IARC), Lyon, France [29,30], adapted for Switzerland by the Federal Food Safety and Veterinary Office (FSVO), Bern, Switzerland). A picture book with portion sizes of 119 common foods and about 60 household measures were utilized to permit more exact quantification of consumed foods. Dietitians were regularly assessed for compliance with 49 standard operating procedures. After collection, the data was screened for inconsistencies according to the IARC guidelines [31] using an updated version of GD (0.2015.09.28). Subsequently, food items, recipes, and ingredients were matched to generic products in the Swiss Food Composition Database (version 37) based on the most relevant nutrient [32]. The corresponding data subset was used for the analyses in this study.

### 2.3. Sugar-Sweetened Beverages Intake

SSB intake was defined as intake of beverages that typically contain free sugars, including soft drinks, energy drinks, isotonic drinks, and fruit juices, even if not additionally sweetened. Artificially sweetened beverages and alcoholic beverages were excluded. This is consistent with the calculations of SSBs in the AHEI-2010 [15], which was used in our study to assess diet quality. For each participant and each 24HDR, the amount of sugar consumed from SSBs was determined and converted to kilocalories (kcal) using the standard Atwater conversion factor of 4 kcal per gram of carbohydrate [33]. Afterwards, the energy contribution of SSBs was calculated by dividing the amount of energy intake from SSBs by the total energy intake. Subsequently, the mean energy contribution of both 24HDR was determined and used to categorize participants into non-, low-, and high-SSB consumers. To define the SSB consumer types, the Swiss dietary guideline was used, which recommends reducing free sugar intake to ≤10% of total daily energy intake [34]. These recommendations are based on the tolerable upper intake level for dietary sugars defined by the European Food Safety Authority [35]. Consistent with Doherty et al. [18] individuals consuming > 10% of total daily energy as sugars from SSBs were defined as high-SSB consumers, individuals consuming ≥ 1% and ≤10% of total daily energy from SSBs were defined as low-SSB consumers, and individuals consuming < 1% of total daily energy from SSBs were defined as non-SSB consumers.

### 2.4. Diet Quality

The AHEI-2010 rates the following eleven dietary components to assess diet quality: vegetables, fruits, whole grains, SSBs and fruit juices, nuts and legumes, red and processed meat, trans fat, long-chain (*n*-3) fatty acids, polyunsaturated fatty acids, sodium, and alcohol [15]. To avoid circularity in the analysis, we used a modified AHEI-2010 score, hereby referred to as non-SSB diet quality or non-SSB AHEI. The non-SSB AHEI score was calculated according to the AHEI-2010 scoring method [15], but excluding the component SSBs and fruit juices. The score of each component ranges from 0 to 10 points. With a total of ten components, the non-SSB AHEI score ranges from 0 to 100 points, with higher scores indicating better diet quality.

### 2.5. Sociodemographic and Anthropometric Variables

*menuCH* participants filled in a written questionnaire at home, which was checked for completeness by the dietitians on the day of the first 24HDR [36]. Derived from the questionnaire were the variables sex (female, male), age group (18–29, 30–49, 50–65, 66–75), education (primary, secondary, tertiary), nationality (Swiss only, Swiss binational, non-Swiss citizen), gross household income in Swiss Francs (CHF) (<6000 CHF/month, 6000–13,000 CHF/month, >13,000 CHF/month), physical activity (low, moderate, high) and smoking status (current, former, never). The linguistic region was determined using the participants’ home cantons (German-speaking region: Canton Aargau, Basel City, Basel Country, Berne, Lucerne, Zurich, and St. Gallen; French-speaking region: Canton Jura, Neuchâtel, Vaud, and Geneva; Italian-speaking region: Canton Ticino).

Height and weight were measured by trained interviewers during the first 24HDR. From these measurements, body mass index (BMI) was calculated, and participants were categorized according to WHO recommendations as underweight (BMI < 18.5 kg/m^2^), normal (BMI ≥ 18.5, but <25.0 kg/m^2^), overweight (BMI ≥ 25.0, but <30.0 kg/m^2^), or obese (BMI ≥ 30.0 kg/m^2^) [37].

### 2.6. Statistical Analyses

*menuCH* participants’ data were weighted for age, sex, marital status, linguistic region, nationality, and household size, to have a study population representative of the Swiss population [38]. 24HDR data were additionally weighted for seasonality and weekdays.

Baseline characteristics of the study population were analyzed descriptively as absolute numbers, unweighted percentages, weighted percentages, weighted median, and weighted interquartile range (IQR). Multivariable, weighted linear regression models were fitted to investigate the association between SSB consumer type and non-SSB AHEI. For each *menuCH* participant, the mean non-SSB AHEI score across the two 24HDR was used as a continuous outcome variable. The variable of interest, SSB consumer type, was defined as a categorical variable with three levels: non-consumer, low-consumer (reference group), and high-consumer. Model 1 (LM1) was adjusted for the participants’ sex (male, female), age group (18–29, 30–49, 50–65, 66–75 years), linguistic region (German, French, Italian), nationality (Swiss only, Swiss binational, non-Swiss citizen), education level (primary school or no degree, secondary, tertiary), household income (<6000, 6000–13,000, >13,000 CHF/month), BMI (underweight, normal, overweight, obese), physical activity (low, moderate, high), and smoking status (never, former, current). Model 2 (LM2) was additionally adjusted for the participants’ average daily energy intake (kcal). Based on LM2, the geometric mean and the corresponding 95% confidence intervals (95% CI) of the non-SSB AHEI scores were determined for the overall study population, by linguistic region, and by age group.

In a sensitivity analysis, the definition of SSB intake was expanded to include artificially sweetened beverages, and the linear regression models were refitted to assess the robustness of the main results. Components of the non-SSB AHEI stratified by SSB consumer type were analyzed descriptively and visualized using boxplots.

Missing values for physical activity (*n* = 524), income (*n* = 585), education (*n* = 3), and smoking status (*n* = 4) were imputed using multivariate imputation by chained equations (MICE) [39]. In total, 25 imputed data sets were generated, and their results were pooled using Rubin’s rule [40]. For all analyses, the R Statistical Software (version 4.4.0) [41] was used, and a statistical significance level of <0.05 was defined.

## 3. Results

Out of 2057 *menuCH* participants, 886 (43%) were non-SSB consumers, 956 (47%) were low-SSB consumers, and 215 (10%) were high-SSB consumers. Non-SSB consumers were more likely to be female, between 66 and 75 years old, and from the Italian-speaking region. High-SSB consumers were mostly male, between 18 and 29 years old, and from the German-speaking region (Table 1).

The fully adjusted linear model (LM2) showed that non-SSB consumers had a higher non-SSB AHEI score than low-SSB consumers by 2.30 points (95% CI 1.32, 3.28). Conversely, high-SSB consumers had a lower non-SSB AHEI compared to low-SSB consumers by 3.16 points (95% CI −4.66, −1.65). The reported findings based on LM2 align with those observed based on the slightly less adjusted LM1 (Table 2).

**Figure 1:**
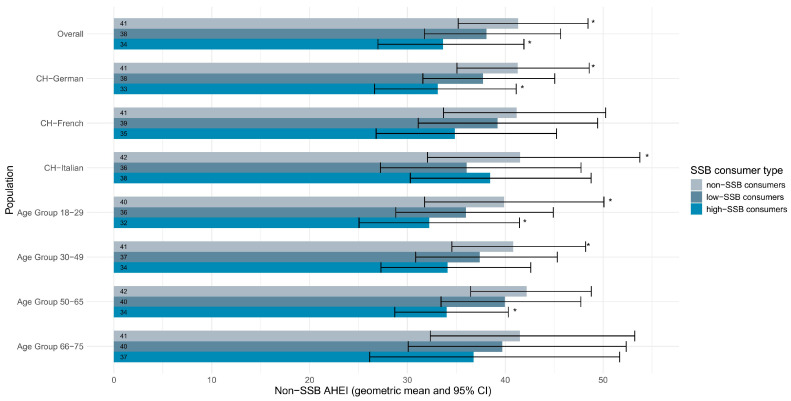
Non-SSB AHEI scores of SSB consumers overall, by linguistic region and age group (geometric mean and 95% CI based on linear regression model [LM2]). * Statistically significant difference compared to low-SSB consumers (ref.).

Non-SSB consumers scored 41 non-SSB AHEI points whereas low- and high-SSB consumers scored 38 and 34 points, respectively (Figure 1). Non-SSB consumers had an AHEI score seven points higher than high-SBB consumers.

In the German-speaking region, the differences in non-SSB AHEI geometric means between consumer types were similar compared to the overall *menuCH* population, with non-SSB consumers having significantly higher, and high-SSB consumers having significantly lower non-SSB AHEI scores (41 and 33 points; Figure 1). No statistically significant differences in the non-SSB AHEI scores were observed in any of the consumer types among participants of the French-speaking region. Non-SSB consumers in the Italian-speaking region had a statistically significantly higher non-SSB AHEI compared to low-SSB consumers.

Participants aged 18–29 years had statistically significant differences in non-SSB AHEI geometric means similar to the overall *menuCH* population (Figure 1). In this age group, non-SSB AHEI scores were significantly higher in non-SSB consumers (40 points) and significantly lower in high-SSB consumers (32 points) compared to low-SSB consumers (36 points). No statistically significant non-SSB AHEI differences among SSB consumer types were observed in the 66–75 years age group. However, in participants aged 30–49 years, non-SSB consumers had a statistically significantly higher non-SSB AHEI compared to low-SSB consumers (41 and 37 points). In participants aged 50–65 years, high-SSB consumers had a statistically significant lower non-SSB AHEI score compared to low-SSB consumers (34 and 40 points). These results remained largely unchanged in the sensitivity analysis including artificially sweetened beverages. The only exception was observed in the subgroup of participants aged 50–65 years, in which non-SSB consumers had a statistically significant higher non-SSB AHEI score compared to low-SSB consumers (42 and 40 points; Table S1 and Figure S2). No clear differences between components of the non-SSB AHEI and the SSB consumer types were observed. A minor trend for higher median AHEI scores among non-SSB consumers compared to low- and high-SSB consumers was observed for the AHEI food components vegetables, fruits, meat, polyunsaturated fatty acids (PUFA), and whole grains (Figure S3).

## 4. Discussion

In this study, we investigated the association of SSB consumer types with non-SSB diet quality using *menuCH* survey data. Overall, higher SSB intake was significantly associated with lower non-SSB diet quality. However, even non-SSB consumers scored a relatively low non-SSB AHEI geometric mean, indicating that the general Swiss diet was of moderate quality. This suggests that a reduction in SSB intake alone is not sufficient to achieve a healthy diet.

A similar US study based on data from the National Health and Nutrition Examination Survey (NHANES) investigated young adults (ages 18–29 years) and observed that 20% of the study population were non-SSB consumers, 48% low-SSB consumers, and 32% high-SSB consumers [18]. In the present study, the distribution of SSB consumer types in participants aged 18–29 years was different (29%, 50%, and 21%, respectively). In both studies, the defined thresholds of sugar intake to classify consumer types were based on dietary sugar recommendations for the whole diet rather than just SSB intake. Notably, Doherty et al. did not include fruit juices in their definition of SSBs [18], which would likely have resulted in even larger differences in high-SSB consumers. US adults consume 145 kcal/day from SSBs [42]. Using the US SSB definition and a typical caloric density of 0.4 kcal/gram [32], Swiss intake would correspond to 96 kcal/day [26]. This suggests that overall SSB intake is approximately 1.5 times higher in the US, which may partially explain the observed differences in consumer type distribution.

To the best of our knowledge, the study from the US [18] is the only other study investigating the association of SSB intake with non-SSB diet quality in adults. Although they used a different dietary index, the findings align with those of our study, indicating that lower SSB intake is associated with higher non-SSB healthy eating index (HEI) scores [18]. The components and score calculations of the HEI differ from the herein used AHEI. However, both diet quality indices are strongly associated with chronic disease risk [15,43,44,45]. Another study examining SSB intake and the HEI in US children found that 25% were non-SSB consumers and 23% were high-SSB consumers [46]. In these children, higher SSB intake was consistently associated with lower diet quality [46]. Differences in age groups, definitions of SSBs, thresholds of consumer types, and diet quality scores limit comparability to the present study. One US randomized controlled trial concluded that a reduction in SSB intake can lead to significantly improved total HEI scores. Over a six month period, intake of sweetened juice-drinks decreased while water and total vegetable intake increased in the intervention group, thus improving the HEI component “empty calories” [47]. Public health interventions targeting reduced SSB intake could therefore not only decrease sugar intake but also improve non-SSB diet quality, providing a twofold benefit [18,47].

Among the three linguistic regions, geometric means of non-SSB AHEI scores were within a comparable range. However, differences in the association between SSB intake and diet quality may reflect underlying cultural and dietary patterns. In the German-speaking region, non-SSB consumers had significantly higher non-SSB diet quality scores than low-SSB consumers, which may be related to higher SSB intake and less favorable fat intake patterns reported in this region [26]. In contrast, no significant differences across SSB consumer types were observed in the French-speaking region. This may reflect higher baseline diet quality, including higher vegetable intake [26,48], which may obscure observable associations. In the Italian-speaking region, high-SSB consumers had higher non-SSB AHEI scores than low-SSB consumers. This finding may be explained by greater adherence to Mediterranean-style dietary patterns, which align closely with AHEI components [48], allowing SSB intake to coexist with otherwise high diet quality. However, the small sample size in the Italian-speaking region limits the interpretation of these findings.

Younger adults were more frequently high-SSB consumers than older adults. This aligns with the findings of previous studies analyzing SSB intake [21,49,50]. Evidence from a systematic review suggests that only small, non-significant declines in SSB intake occur between ages 18 and 30, supporting the persistence of high-sugar consumption habits during early adulthood [51]. From a life-course perspective, young adulthood may therefore represent a critical period for the establishment of dietary behaviors, with early high intake potentially contributing to cumulative long-term health risks [52]. In our study population, the proportion of high-SSB consumers was lower among participants with higher education level, suggesting that social determinants may also contribute to age-related differences in SSB intake. Structural public health measures, such as pricing policies or marketing regulations, may therefore be particularly relevant for younger adults, who may be more sensitive to environmental and economic influences. This supports prioritizing young adulthood in population-level strategies aimed at reducing SSB intake.

In Switzerland, trends of SSB intake over time are not available as of now, since the *menuCH* survey was the first of its kind. However, a review showed a 23% decrease in SSB intake in high-income countries between 2005 and 2018 [21]. If this were the case for Switzerland, future research could investigate whether decreasing SSB intake has a causal relationship with the non-SSB diet quality. However, it is important to consider long-term trends in the non-SSB diet as well. In one of our previous publications, we investigated adherence to the cancer-prevention recommendations proposed by the World Cancer Research Fund/American Institute for Cancer Research. Based on data from six Swiss Health Surveys (1992–2017), we observed a continuous decrease in adherence to the fruit and vegetable recommendations from 2007 onwards and no major changes in red and processed meat consumption [53]. These findings suggest that even if SSB intake decreases, the overall quality of the non-SSB diet may only improve if adherence to other dietary recommendations does not worsen over time.

The non-SSB AHEI scores observed in our population were generally relatively low. Although the AHEI was originally developed in the US context, standard AHEI scores of Swiss adults are comparable to those reported in the US, UK, and China [48], and the AHEI has been successfully applied in prior Swiss and global studies [22,48,54,55]. This suggests that, despite differences in local dietary habits, the AHEI remains a useful tool for comparing overall dietary patterns in Swiss populations.

The AHEI component analysis showed that the calculated non-SSB AHEI scores by component were generally similar among all SSB consumer types with only a minor trend for higher median AHEI scores among non- compared to low- and high-SSB consumers for the fruits, meat, PUFA, vegetables, and whole grains food components. Therefore, instead of specific dietary recommendations for certain SSB consumer types, it may be more effective to focus on general dietary improvements to enhance non-SSB diet quality in the population.

Our study has some limitations. With a cross-sectional design, the *menuCH* survey was observational and therefore cannot show causal relationships between SSB intake and non-SSB diet quality. Data were collected from 2014–2015. Although more than a decade has passed, the absence of major regulatory policies, such as sugar taxes, suggests that overall dietary habits in Switzerland have remained largely stable. However, evidence from European countries indicates a shift from sugar-sweetened soft drinks toward reduced- or no-sugar alternatives, suggesting that current SSB intake in Switzerland may be lower than observed in this study [56]. Participants voluntarily joined the *menuCH* survey and therefore may have been more health-conscious than non-participants. SSB intake may have been underreported due to recall and reporting bias [57,58]. In the absence of more precise guidelines, the defined thresholds of sugar intake to classify consumer types were based on dietary sugar recommendations for the whole diet rather than just SSB intake [35]. Although this is consistent with previous research [18], it may have led to an underestimation of low- and high-SSB consumers. In addition, whilst the recommendations are based on free sugar intake [35], the thresholds of sugar intake in this study were based on total sugar intake from SSBs, as information on free sugar content was not available in the Swiss Food Composition Database. However, based on a previous analysis using *menuCH* data [24], total and free sugar contents in the beverages included in our study are similar. The sample sizes of certain subgroups were low, which may have caused random variations in the results. Furthermore, the AHEI-2010 only includes selected components, which are assumed to be of equal importance to each other. These components cannot take the complexity of a whole diet into account [59,60]. Future research could focus on refining dietary scoring systems by incorporating weighting schemes that reflect the impact of specific dietary components on health outcomes. In LM2, average daily energy intake may partially mediate the association between SSB intake and non-SSB diet quality, which could result in overadjustment. Lastly, residual confounding remains possible.

Several strengths are noteworthy. The *menuCH* survey was intended to be representative of the Swiss population. Two non-consecutive 24HDRs were conducted with trained dietitians and assistive equipment. The analysis was controlled for anthropometric, sociodemographic, and lifestyle factors. To the best of our knowledge, this is the first study that examined the association of SSB intake with non-SSB diet quality in a European population.

## 5. Conclusions

Lower SSB intake was associated with higher non-SSB diet quality, suggesting that those with less SSB intake made healthier food choices for the rest of their diet. This association was more apparent in young adults and individuals from the German-speaking region of Switzerland. Overall, non-SSB diet quality remained moderate, even among non-SSB consumers. Prospective studies are needed to clarify which alternative food choices individuals make when reducing SSB intake. These findings highlight the importance of promoting overall healthy dietary patterns, including components of the AHEI such as vegetables, fruits, whole grains, and unsaturated fats. Public health authorities should consider strategies that both reduce SSB intake and support improvements in the overall diet to help lower chronic disease risk in Switzerland.

## Figures and Tables

**Table 1:** Sociodemographic, anthropometric, and lifestyle characteristics of Swiss adults from the *menuCH* survey conducted in 2014 and 2015, stratified by SSB consumer type (*n* = 2057).

Variable ^1^	Overall ^2^	SSB Consumer Type ^3^		
		Non-Consumer	Low-Consumer	High-Consumer
N (%)	2057 (100.0)	886 (43.1)	956 (46.5)	215 (10.4)
Sex, *n* (%, %*)				
Male	933 (45.4, 49.8)	373 (40.0, 37.6)	448 (48.0, 49.0)	112 (12.0, 13.4)
Female	1124 (54.6, 50.2)	513 (45.6, 46.3)	508 (45.2, 44.1)	103 (9.2, 9.6)
Age group, *n* (%, %*)				
18–29	400 (19.4, 18.8)	113 (28.3, 28.9)	210 (52.5, 50.4)	77 (19.3, 20.7)
30–49	765 (37.2, 40.1)	303 (39.6, 39.2)	377 (49.3, 49.0)	85 (11.1, 11.8)
50–65	588 (28.6, 28.2)	296 (50.3, 48.0)	255 (43.4, 44.2)	37 (6.3, 7.7)
66–75	304 (14.8, 12.9)	174 (57.2, 56.3)	114 (37.5, 38.4)	16 (5.3, 5.3)
Linguistic region ^4^, *n* (%, %*)				
German	1341 (65.2, 68.8)	546 (40.7, 39.7)	639 (47.7, 47.5)	156 (11.6, 12.8)
French	502 (24.4, 25.7)	221 (44.0, 44.0)	237 (47.2, 46.8)	44 (8.8, 9.2)
Italian	214 (10.4, 5.6)	119 (55.6, 60.4)	80 (37.4, 33.2)	15 (7.0, 6.4)
Nationality, *n* (%, %*)				
Swiss only	1492 (72.5, 60.6)	643 (43.1, 41.2)	693 (46.4, 47.5)	156 (10.5, 11.4)
Swiss binational	297 (14.4, 14.4)	124 (41.8, 43.7)	148 (49.8, 48.7)	25 (8.4, 7.6)
Non-Swiss citizen	268 (13.0, 25.0)	119 (44.4, 42.9)	115 (42.9, 43.0)	34 (12.7, 14.1)
Education (highest degree), *n* (%, %*)				
Primary school or no degree	89 (4.3, 4.6)	37 (41.6, 43.0)	39 (43.8, 44.8)	13 (14.6, 12.2)
Secondary (e.g., apprenticeship)	968 (47.1, 43.1)	422 (43.6, 41.3)	434 (44.8, 45.3)	112 (11.6, 13.4)
Tertiary (e.g., university)	997 (48.5, 52.0)	427 (42.8, 42.7)	481 (48.2, 47.7)	89 (8.9, 9.6)
Missing	3 (0.1, 0.3)	0 (0.0, 0.0)	2 (66.7, 43.5)	1 (33.3, 56.5)
Household income (gross), *n* (%, %*)				
<6000 CHF/month	346 (16.8, 17.6)	152 (43.9, 42.7)	161 (46.5, 46.4)	33 (9.5, 10.9)
6000–13,000 CHF/month	841 (40.9, 39.4)	365 (43.4, 41.8)	390 (46.4, 46.7)	86 (10.2, 11.6)
>13,000 CHF/month	285 (13.9, 14.7)	120 (42.1, 40.0)	137 (48.1, 50.5)	28 (9.8, 9.6)
Missing	585 (28.4, 28.3)	249 (42.6, 42.8)	268 (45.8, 44.4)	68 (11.6, 12.8)
BMI categories, *n* (%, %*)				
Underweight	51 (2.5, 2.3)	15 (29.4, 33.1)	29 (56.9, 51.1)	7 (13.7, 15.9)
Normal	1115 (52.2, 52.4)	472 (42.3, 41.6)	527 (47.3, 47.0)	116 (10.4, 11.4)
Overweight	629 (30.6, 30.7)	273 (43.4, 41.9)	288 (45.8, 46.7)	68 (10.8, 11.4)
Obese	262 (12.7, 12.5)	126 (48.1, 45.6)	112 (42.7, 43.2)	24 (9.2, 11.2)
Physical activity, *n* (%, %*)				
Low	219 (10.7, 11.2)	104 (47.5, 47.5)	93 (42.5, 41.0)	22 (10.0, 11.5)
Moderate	487 (23.7, 23.8)	215 (44.1, 44.7)	235 (48.3, 47.2)	37 (7.6, 8.1)
High	827 (40.2, 40.2)	351 (42.4, 40.4)	383 (46.3, 46.7)	93 (11.2, 12.9)
Missing	524 (25.5, 24.8)	216 (41.2, 39.3)	245 (46.8, 48.1)	63 (12.0, 12.6)
Smoking status, *n* (%, %*)				
Never	914 (44.4, 42.0)	387 (42.3, 42.0)	432 (47.3, 47.4)	95 (10.4, 10.6)
Former	688 (33.4, 35.4)	322 (46.8, 44.8)	313 (45.5, 45.8)	53 (7.7, 9.5)
Current	451 (21.9, 22.4)	177 (39.2, 38.1)	208 (46.1, 45.9)	66 (14.6, 15.9)
Missing	4 (0.2, 0.3)	0 (0.0, 0.0)	3 (75.0, 52.5)	1 (25.0, 47.5)
Daily energy intake ^5^ (kcal), *median*, [*IQR*]	2088 [1666, 2551]	1953 [1565, 2356]	2179 [1770, 2661]	2184 [1693, 2741]
Non-SSB AHEI ^6^, *median*, [*IQR*]	38.3 [31.3, 46.0]	40.4 [33.1, 48.3]	37.4 [30.6, 45.2]	34.6 [27.9, 40.1]

^1^ Categorical variables are depicted as unweighted absolute numbers (*n*), unweighted percentages (%), and weighted percentages (%*). Continuous variables are depicted as weighted median and interquartile range [*IQR*]. The data of the participants were weighted based on the *menuCH* weighting strategy for sex, age, marital status, major living region in Switzerland, nationality, household size, and weekday and season of the recall day [38]. ^2^ Participants (%) sum to total participants (100%) within each variable. ^3^ Participants (%) sum to total participants (100%) within each variable category across SSB consumer type. Non-SSB consumers had a mean sugar intake from SSBs of <1% of total daily energy intake, low-SSB consumers had a mean sugar intake from SSBs between ≥1% and ≤10% of total daily energy intake, and high-SSB consumers had a mean sugar intake from SSBs of >10% of total daily energy intake. ^4^ German-speaking region: Canton Aargau, Basel City, Basel Country, Berne, Lucerne, Zurich, and St. Gallen); French-speaking region: Canton Jura, Neuchâtel, Vaud, and Geneva; Italian-speaking region: Canton Ticino. ^5^ For each *menuCH* participant, average daily energy intake was calculated based on two 24 h dietary recalls. The median and interquartile range of the average daily energy intake were then determined for the overall study population and stratified by SSB consumer type. ^6^ The non-SSB AHEI was calculated using ten components of the AHEI-2010 [15], namely vegetables, fruits, whole grains, nuts, meat, trans fat, long chain omega-3 fatty acids, polyunsaturated fatty acids, sodium, and alcohol. The higher the score, the better the diet quality. Depicted is the mean non-SSB AHEI score based on two 24 h dietary recalls for each *menuCH* participant.

**Table 2:** Point estimates and 95% confidence intervals of the linear regression model investigating the association between SSB consumer type and non-SSB AHEI in the *menuCH* study population (*n* = 2057, Switzerland).

Variable	Non-SSB AHEI (LM1 ^1,2^)		Non-SSB AHEI (LM2 ^1,3^)	
	β	95% CI	β	95% CI
SSB consumer type				
Non-consumer	2.62	1.64, 3.60	2.30	1.32, 3.28
Low-consumer (ref.)	1.00		1.00	
High-consumer	−3.16	−4.77, −1.74	−3.16	−4.66, −1.65

^1^ Linear regression models were fitted. The estimates were weighted based on the *menuCH* weighting strategy for sex, age, marital status, major living region in Switzerland, nationality, household size, and weekday and season of the recall day [38]. ^2^ LM1 was adjusted for sex, age group, linguistic region, nation group, education, income, physical activity, smoking status, and BMI group. ^3^ LM2 was identical to LM1 but additionally adjusted for daily energy intake.

## Data Availability

The data and further documents of the *menuCH* study are available by request under https://menuch.iumsp.ch.

## Supplementary Materials

The following supporting information can be downloaded at: https://www.mdpi.com/article/10.3390/nu18050718/s1, Figure S1: Participant flowchart of the menuCH survey (*n* = 2057); Table S1: Sensitivity analysis of the association of SSB consumer type with non-SSB AHEI in the Swiss population (*n* = 2057), including artificially sweetened; Figure S2: Sensitivity analysis of the non-SSB AHEI scores of SSB consumers overall, by linguistic region and age group including artificially sweetened beverages; Figure S3: Weighted mean and interquartile range of the average non-SSB AHEI component score of *menuCH* participants (*n* = 2057).

## Abbreviations

The following abbreviations are used in this manuscript:

24HDR	24 h dietary recalls
AHEI	Alternative healthy eating index
BMI	Body mass index
CHF	Swiss francs
CI	Confidence intervals
CVD	Cardiovascular diseases
FSVO	Federal Food Safety and Veterinary Office
GBD	Global burden of disease
GD	GloboDiet^®^
HEI	Healthy eating index
IARC	International Agency for Research on Cancer
kcal	Kilocalories
IQR	Interquartile range
LM1	Linear model 1
LM2	Linear model 2
*menuCH*	Swiss National Nutrition Survey
MICE	Multivariate Imputation by Chained Equations
NHANES	National Health and Nutrition Survey
NCDs	Non-communicable diseases
SSB	Sugar-sweetened beverages
WHO	World Health Organization
