# Supplementary material for: Sugar-Sweetened Beverage (SSB) Intake Is Associated with Non-SSB Diet Quality in Swiss Adults
Source: Nutrients. 2026 Feb 24;18(5):718. doi: 10.3390/nu18050718 (PMC12987221; doi:10.3390/nu18050718)

Sugar-sweetened beverage (SSB) intake is associated with non-SSB diet quality in Swiss adults
Lukas Abraham, Flurina Suter, Giulia Pestoni, Sabine Rohrmann

Figure S1: Participant flowchart of the *menuCH* survey (n = 2057).

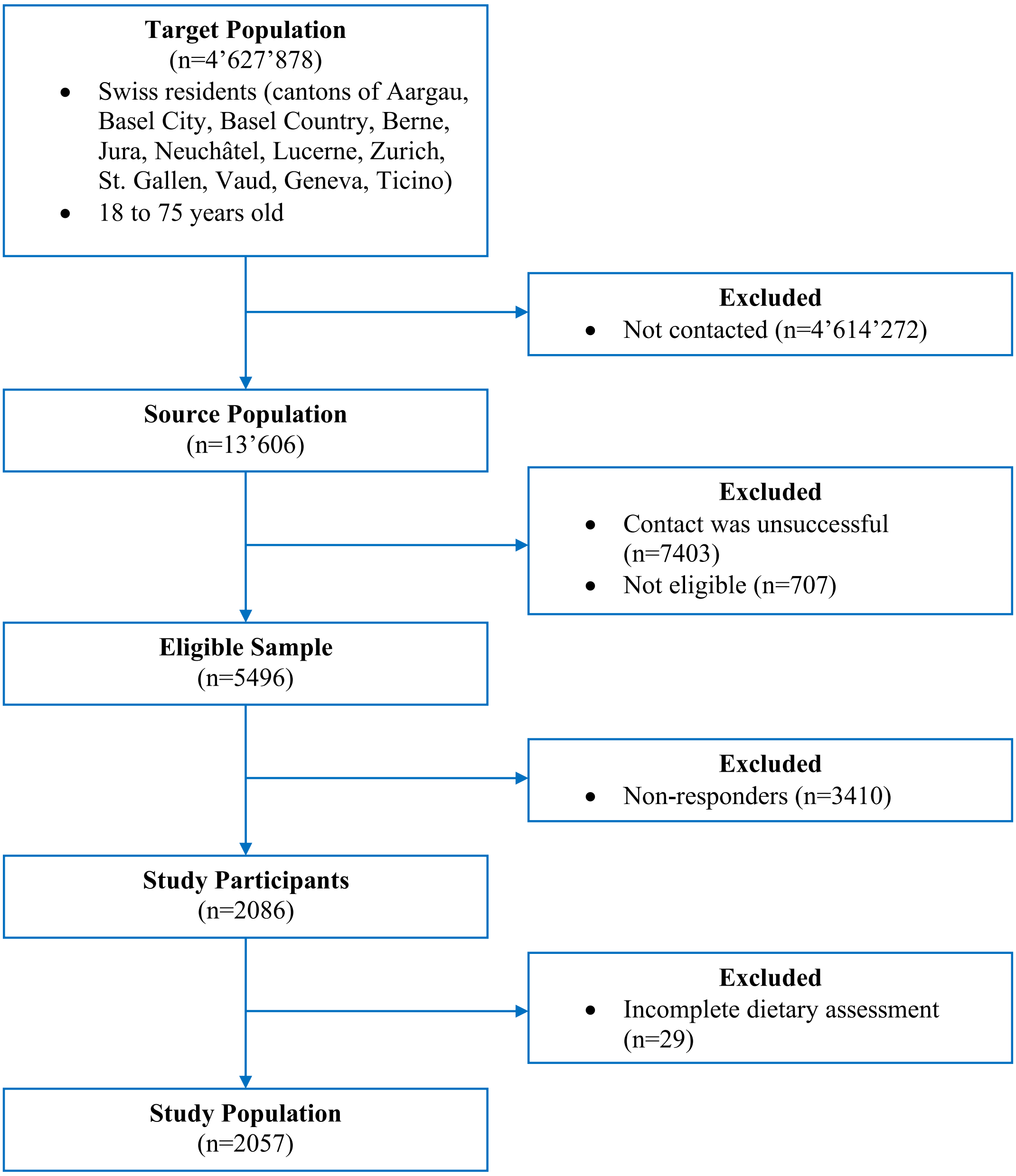

Table S1: Sensitivity analysis of the association of SSB consumer type with non-SSB AHEI in the Swiss population (n=2057), including artificially sweetened beverages in the definition of SSB intake.

	**Non-SSB AHEI (LM1[1,2])**		**Non-SSB AHEI (LM2[1,3])**	
**Variable**	**β**	**95% CI**	**β**	**95% CI**
SSB consumer type[4]				
Non-consumer	2.65	1.67, 3.63	2.34	1.36, 3.32
Low-consumer (ref.)	1.00		1.00	
High-consumer	-3.25	-4.76, -1.74	-3.16	-4.66, -1.66

SSB, sugar-sweetened beverages; AHEI, alternate healthy eating index; LM1, linear model 1; LM2, linear model 2; CI, confidence interval.

[1]Linear regression models were fitted. The estimates were weighted based on the *menuCH* weighting strategy for sex, age, marital status, major living region in Switzerland, nationality, household size, and weekday and season of the recall day [(36)].

[2]LM1 was adjusted for sex, age group, linguistic region, nation group, education, income, physical activity, smoking status, and BMI group.

[3]LM2 was identical to LM1 but additionally adjusted for daily energy intake.

[4]Participants of the *menuCH* survey were categorized into SSB consumer types. Non-consumers had an average SSB sugar intake of <1% of total daily energy intake, low-consumers had an average SSB sugar intake between ≥1% and ≤10% of total daily energy intake, and high-consumers had an average SSB sugar intake of >10% of total daily energy intake.

Figure S2: Sensitivity analysis of the non-SSB AHEI scores of SSB consumers overall, by linguistic region and age group (geometric mean and 95% CI based on linear regression model [LM2]), including artificially sweetened beverages in the definition of SSB intake.
* Statistically significant difference compared to low-SSB consumers (ref.)

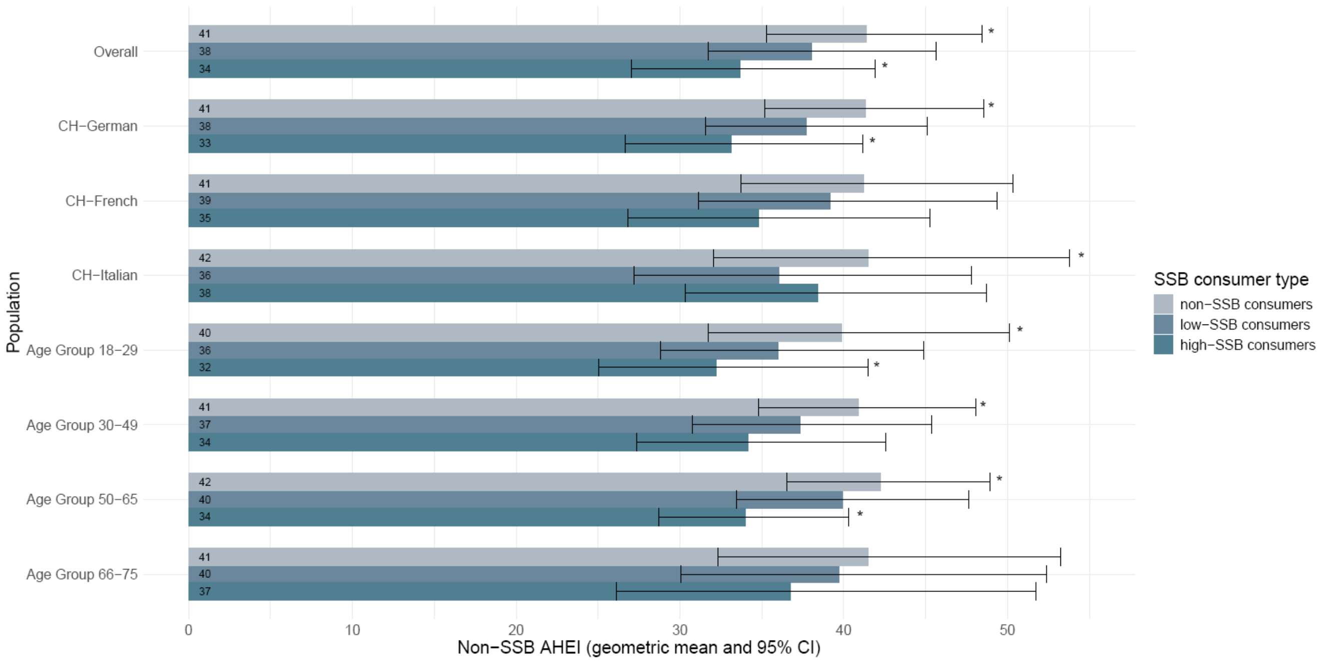

Figure S3: Weighted mean and interquartile range of the average non-SSB AHEI component score of *menuCH* participants (n=2057), stratified by SSB consumer type. Non-SSB consumers had a mean sugar intake from SSB of <1% of total daily energy intake, low-SSB consumers had a mean sugar intake from SSB between ≥1% and ≤10% of total daily energy intake and high-SSB consumers had a mean sugar intake from SSB of >10% of total daily energy intake.
SSB, sugar-sweetened beverages; AHEI, alternate healthy eating index.

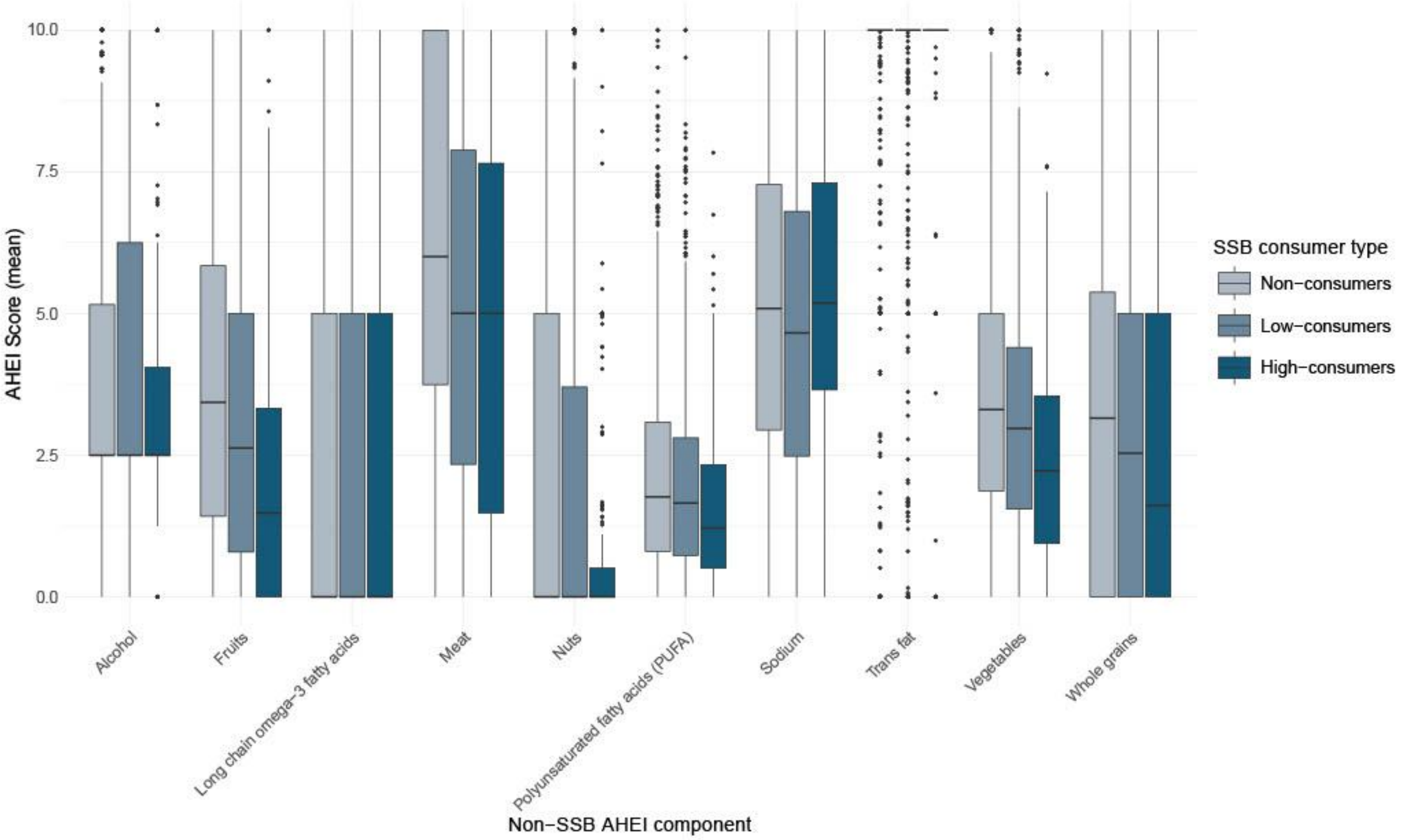

Supplement: Supplementary file 1 [file nutrients-18-00718-s001.zip › nutrients-4136179-supplementary.pdf]
